# Identification and Functional Annotation of Genes Differentially Expressed in the Reproductive Tissues of the Olive Tree (*Olea europaea* L.) through the Generation of Subtractive Libraries

**DOI:** 10.3389/fpls.2017.01576

**Published:** 2017-09-13

**Authors:** Adoración Zafra, Rosario Carmona, José A. Traverso, John T. Hancock, Maria H. S. Goldman, M. Gonzalo Claros, Simon J. Hiscock, Juan D. Alche

**Affiliations:** ^1^Plant Reproductive Biology Laboratory, Department of Biochemistry, Cellular and Molecular Biology of Plants, Estación Experimental del Zaidín, Consejo Superior de Investigaciones Científicas Granada, Spain; ^2^Faculty of Health and Life Sciences, University of the West of England Bristol, United Kingdom; ^3^Departamento de Biologia, Faculdade de Filosofia, Ciências e Letras de Ribeirão Preto, Universidade de São Paulo São Paulo, Brazil; ^4^Departamento de Biología Molecular y Bioquímica, Universidad de Málaga Málaga, Spain; ^5^School of Biological Sciences, University of Bristol Bristol, United Kingdom

**Keywords:** gynoecium, leaf, olive, pollen, self-incompatibility, SSH, transcripts

## Abstract

The olive tree is a crop of high socio-economical importance in the Mediterranean area. Sexual reproduction in this plant is an essential process, which determines the yield. Successful fertilization is mainly favored and sometimes needed of the presence of pollen grains from a different cultivar as the olive seizes a self-incompatibility system allegedly determined of the sporophytic type. The purpose of the present study was to identify key gene products involved in the function of olive pollen and pistil, in order to help elucidate the events and signaling processes, which happen during the courtship, pollen grain germination, and fertilization in olive. The use of subtractive SSH libraries constructed using, on the one hand one specific stage of the pistil development with germinating pollen grains, and on the other hand mature pollen grains may help to reveal the specific transcripts involved in the cited events. Such libraries have also been created by subtracting vegetative mRNAs (from leaves), in order to identify reproductive sequences only. A variety of transcripts have been identified in the mature pollen grains and in the pistil at the receptive stage. Among them, those related to defense, transport and oxidative metabolism are highlighted mainly in the pistil libraries where transcripts related to stress, and response to biotic and abiotic stimulus have a prominent position. Extensive lists containing information as regard to the specific transcripts determined for each stage and tissue are provided, as well as functional classifications of these gene products. Such lists were faced up to two recent datasets obtained in olive after transcriptomic and genomic approaches. The sequences and the differential expression level of the SSH-transcripts identified here, highly matched the transcriptomic information. Moreover, the unique presence of a representative number of these transcripts has been validated by means of qPCR approaches. The construction of SSH libraries using pistil and pollen, considering the high interaction between male-female counterparts, allowed the identification of transcripts with important roles in stigma physiology. The functions of many of the transcripts obtained are intimately related, and most of them are of pivotal importance in defense, pollen-stigma interaction and signaling.

## Introduction

The olive (*Olea europaea* L.) is an important crop in Mediterranean countries. The fruit is used for the production of olive oil. Olive oil yield, organoleptic properties, quality, fatty acid content and many other parameters are highly dependent on the procedures used for olive oil production, including which olive cultivars are used. Asexual propagation of this tree, achieved by different methods (Böhm, 2013), is the usual practice since its domestication. This practice results in very high heteroplasmy, as assessed by the accumulation of mutations in a non-coding sequence of the mitochondrial genome when vegetative propagation is maintained for a long period of time (García-Díaz et al., 2003). However, olive production relies on the successful achievement of sexual reproduction. This plant has been suggested to harbor a self-incompatibility system of the gametophytic type (Cuevas and Polito, 1997; Ateyyeh et al., 2000; Wu et al., 2002), as described for the Oleaceae family (Igic and Kohn, 2001). However, most recent and abundant literature on the issue demonstrates that the self-incompatibility in olive is sporophytic (Kusaba et al., 2001; Allen et al., 2010a; Breton et al., 2014, 2016, 2017; Farinelli et al., 2015; Saumitou-Laprade et al., 2017). The fine mechanisms governing this system are currently being deciphered, and are likely to explain the divergence of incompatibility mechanisms which can occur among members of the same family, as seen in Arabidopsis (Kusaba et al., 2001). The SI mechanism described in the olive involves the preferential presence of pollen grains from a different cultivar for successful fertilization (allogamy). The main worry of the growers is the yield, which is affected by the pollinisers–pollinator relationship (Breton and Bervillé, 2012). In the case of the olive, wind is the main factor affecting the yield, as the dispersion of the pollen in olive is mainly anemophylous.

The use of high-throughput analytic methods based in next-generation sequencing (NGS) is rapidly reaching the study of the olive tree. A number of recent studies have described the generation of several olive transcriptomes (reviewed by Muleo et al., 2016), which have been generated from different organs and adaptive responses, sometimes discriminating their varietal origin and built preferentially by pyrosequencing/Illumina sequencing and array technologies. Thus, transcriptomes have been generated to approach flower and fruit development (Alagna et al., 2009, 2016; Galla et al., 2009; Ozgenturk et al., 2010; Muñoz-Mérida et al., 2013; Carmona et al., 2015; Iraia et al., 2016), fruit abscission (GilAmado and Gomez-Jimenez, 2012; Parra et al., 2013), abiotic stress responses (Bazakos et al., 2015; Guerra et al., 2015; Leyva-Pérez et al., 2015), miRNA (Donaire et al., 2011; Yanik et al., 2013), plant architecture (González-Plaza et al., 2016), and even comparative transcriptomics (Sarah et al., 2017). Genome sequence of the olive tree, corresponding to 95–99% of the estimated genome length was recently obtained and annotated by Cruz et al. (2016). Such annotation was assisted by RNAseq from different tissues and stages, and represents an important resource for future research on olive tree, as well as for breeding purposes.

The present study was based on the construction of several cDNA libraries that were subtracted using the SSH method. The aim was to study the reproductive biology of the olive and particularly to obtain clues regarding pollen and stigma physiology, including the presence of differentially expressed enzymes, allergens and other relevant gene products. For that purpose we used reproductive tissues (pollen and pistil) as well as vegetative tissues (leaf as the subtractive item). For each pair combination, the forward and reverse libraries were constructed.

## Materials and methods

### Plant material

The different tissues were obtained from adult olive trees (*Olea europaea*, cv. Picual) growing at the Estación Experimental del Zaidín (Granada, Spain). Pistils were excised from the complete flower at the stage of development 4, dehiscent anthers, as defined by Zafra et al. (2010). These pistils normally include a relatively high number of mature (dehiscent) pollen, hydrated pollen grains, and even germinating pollen grains and pollen tubes, either over the stigma surface or through the transmitting tissues of the style or the ovary. The mature pollen grains were collected during the anthesis period using large paper bags by vigorously shaking the inflorescences. Pollen was sequentially sieved through a mesh in order to separate the grains from the debris. Young leaves were also selected. In all the three cases the different tissues were quickly frozen in liquid nitrogen and stored at −80°C. Samples from three consecutive years were used for the present analysis.

### Construction of the suppression subtractive hybridization (SSH) libraries

Total RNA was isolated using the RNeasy Plant Mini Kit (Qiagen) from samples of the different years, and the contaminating genomic DNA was removed by DNAase I (Qiagen) treatment followed by a clean-up with the RNeasy MinElute Cleanup kit (Qiagen). cDNA was then synthesized from pistil, leaf, and mature pollen total RNA using the SMART PCR cDNA Synthesis kit (Clontech). The subtracted libraries were constructed with the PCR-Select cDNA Subtraction Kit (Clontech). A total of 6 libraries were constructed: 1. Pistil subtracted with pollen [P(Po)]; 2. Pollen subtracted with pistil [Po(P)]; 3. Pistil subtracted with leaf [P(L)]; 4. Leaf subtracted with pistil [L(P)]; 5. Pollen subtracted with leaf [Po(L)]; 6. Leaf subtracted with pollen [L(Po)], according to the manufacturer's instructions. Two rounds of PCR amplifications were also performed according to the manufacturer's protocol in order to enrich differentially regulated genes, by using the PCR Primer 1 and the Nested PCR primer 1 and 2R as indicated in the manufacturer's instructions and provided by the kit.

### Cloning and differential screening

The secondary PCR products were cloned into the T/A cloning vector pGEM-T Easy (Promega) according to the manufacturer's instructions and transformed into DH5α *E. coli* cells. The colonies containing inserts were picked and used as template for PCR. The primers used in this case were SP6 and T7. Sanger sequencing of PCR products was carried out at the Estación Experimental del Zaidín DNA Sequencing Service (CSIC, Granada, Spain), the Laboratório de Biologia Molecular de Plantas (Universidade de São Paulo, Brazil), and other commercially available facilities. With the aim to perform the differential screenings, a number of membrane replicates were prepared, each one containing 1 μl of the PCR product per dot, which were spotted onto nylon membranes and fixed with a brief wash in 2x SSC followed by baking at 120°C during 30 min. The membrane replicates were probed with the forward-subtracted probe, the reverse-subtracted probe, the unsubtracted tester probe, and the unsubtracted driver probe in each case. The labeled probes were generated from the secondary PCRs products described in the (SSH) library construction section, which were purified using the MinElute PCR Purification Kit (Qiagen). DIG-DNA labeling, determination of labeling efficiency, hybridization, and immunological detection were carried out as described in the DIG High Prime DNA Labeling and Detection Starter Kit II (Roche) instruction manual. The membranes were revealed with the CSPD ready-to-use chemiluminescent substrate (Roche), exposed to ChemiDoc XRS system (Bio-Rad). Images were gathered with a supersensitive 12-bit CCD after 30 min of exposition (Supplementary Figure 1). All hybridizations and image captures were repeated twice.

### Sequencing and data analysis

Transcripts were compared (using BLASTn) against non-redundant protein databases at the National Center for Biotechnology Information (http://www.ncbi.nlm.nih.gov; Altschul et al., 1990) (*E*-value 10^−4^) and also against the non-redundant proteins unique transcripts Olea EST database (Alagna et al., 2009). The Blast2Go (http://www.blast2go.com/b2ghome) software was used (Conesa et al., 2005) to carry out the statistical analysis of GO (Gene Ontology) terms. For the analysis of the contigs obtained from the singletons, the Codon Code Aligner software was used (http://www.codoncode.com/aligner/).

The Venn diagrams were constructed using the transcripts of the 6 SSH libraries analyzed. Three groups were considered, corresponding to pollen, pistil and leaf transcripts. The VENNY software (http://bioinfogp.cnb.csic.es/tools/venny/) was used for this purpose. With the aim to compare the output results after the comparison against the NCBI and Olea EST databases, two diagrams were performed separately.

To retrieve the putative Arabidopsis homologs of the olive clones obtained, the sequences from the transcripts of two selected libraries [Po(P) and P(Po)] were compared against the Arabidopsis Information Resource (TAIR) webpage (http://www.arabidopsis.org/Blast/). A BLASTn against the TAIR10 Transcripts (−introns, +UTRs) (DNA) was carried out. The matrix weight was Blosum45, the nucleic mismatch −3, gapped alignments ON. The output results were used as input data in the plant biology resource from Genevestigator (https://www.genevestigator.com/gv/plant.jsp). The anatomy tool from this webpage was used to construct the heatmap representing the level of expression of the transcripts in olive corresponding to defense, oxidative metabolism and transport. These categories were chosen attending to two criteria: firstly a highly represented number of transcripts and also due to the implication in the reproductive process.

### qPCR validation of substractive transcripts

Total RNA from pollen, pistil and leaf from olive cv. Picual were extracted using the RNeasy Plant Mini Kit (Qiagen) according to the manufacturer's instructions from samples obtained after three consecutive years as described above. Two μg of total RNA was reversed transcribed using the High-Capacity cDNA Reverse Transcription kit (Applied Biosystems, Thermo Scientific). Nine independent reverse transcriptase reactions were carried out. cDNA was stored at −20°C until use for qPCR analyses. Primers were designed for transcripts putatively specific of each tissue as determined by the differential screening. The Primer BLAST software (NCBI) was used for primer design with modifications in the default settings [PCR product size: 70–150 bp; Primer Tm 58–62°C: Organism: green plants; Primer size: 18-23 bp; Primer GC content: 30–80%; Hyb Oligo Size: 18–30 bp; Hyb Oligo Tm: 68–72°C; Hyb Oligo GC content: 30–80%]. Putative amplicons were blasted against Reprolive database aimed to confirm the specificity. For details of the primer sequence and expected sizes of the amplicons, see Supplementary Table 1. LightCycler FastStart DNA Master Syber Green (Roche) was used in a Light Cycler 480 Instrument II (Roche Diagnostic, Mannheim, Germany) in a 20 μl reaction volume. Samples were run in duplicate for each experiment. Expression levels of target genes were normalized as regard to the expression level of two housekeeping genes (Zinc-finger and katanin p60) and their relative expression levels were calculated with the ΔΔCt rule (Taylor et al., 2016). Housekeeping genes were gathered based on an automatic screening of the Reprolive database and were previously tested in pollen, pistil and leaf tissues (Carmona et al., 2016, 2017). Housekeeping sequences and amplicon sizes are detailed in Supplementary Table 1.

### Comparative transcriptomics

#### Pre-processing of subtractive sequences

All clones sequenced were pre-processed using SeqTrimNext (Falgueras et al., 2010) as described in Carmona et al. (2015) to remove linkers, adaptors, vector fragments and contaminated sequences among others, while keeping the longest informative part of the sequence. Sequences below 100 bp were discarded.

#### Annotation and correspondence with olive transcriptomes

Useful sequences were annotated using Full-LengtherNext (Seoane et al., in preparation). Additionally, the correspondence of the subtractive sequences with the transcriptomes reported in ReprOlive database (http://reprolive.eez.csic.es; Carmona et al., 2015), as well as with the transcripts deduced from the first olive tree genome draft (89,982 transcripts) (Cruz et al., 2016) were determined. This correspondence was estimated by comparing (using BLASTn, *E*-value 10^−6^) the subtractive sequences against the mentioned transcriptomes. With regard to ReprOlive transcriptomes, sequences from pollen libraries [Po(L) and Po(P)] were compared against the pollen transcriptome (27,823 transcripts), sequences from pistil libraries [P(Po) and P(L)] were compared against the pistil transcriptome (60,400 transcripts), whereas sequences from leaf libraries [L(Po) and L(P)] were compared against the vegetative transcriptome (38,919 transcripts).

#### Presence/absence of subtractive sequences in different stages/tissues

In order to estimate the validity of the subtractive SSH libraries constructed, an *in silico* approximation was performed, taking advantage of the availability of Roche/454 reads used in ReprOlive (Carmona et al., 2015), which belong to seven different tissues and/or developmental stages: leaf, mature pollen, germinated pollen at two different times after hydration (1 and 5 h) and pistil at developmental stages 2, 3, and 4 as defined by Zafra et al. (2010). Each one of the subtractive sequences was compared by BLASTn (*e*-value 1e^−6^) against the reads of each of the seven tissues/stages separately. In the event of at least one read matching with the subtractive sequence (considering a 90% or greater of identity and an alignment of 85% or greater of the subtractive sequence length), the subtractive sequence was considered to be present in this particular tissue/stage (and reported as “YES” in the corresponding output information).

### Availability of data and materials

The datasets supporting the conclusion of this article are available in the European Nucleotide Archive (http://www.ebi.ac.uk/ena/data/view/PRJEB13716) in the fastq.gz format with the title “SSH libraries of the olive tree (*Olea europaea* L.) reproductive tissues.” Fastq identifiers in Supplementary Tables 2–7 allow location of every single sequence in its corresponding fastq file deposited at ENA.

## Results and discussion

Six libraries were generated by using the combination of tester/driver tissues indicated in Table 1. Special attention was given to the P(Po), P(L), and Po(L) libraries. Table 1 includes information as regarded to the number of clones identified and sequenced from each one of the six SSH libraries generated.

**Table 1:** SSH libraries constructed and descriptive parameters about the clones sequenced.

**Name of the library**	**Tester tissue**	**Driver tissue**	**No. Sequenced clones**	**Total ESTs**	**Total contigs**	**Mean contig(bp)**	**Mean ESTs (bp)**	**% Redundancy**	**BLAST Hits(%) (NCBIdb)**	**BLAST Hits(%) (OLEA ESTdb)**
Po(P)	Pollen	Pistil	288	127	46	588	440	3.17	76	90
P(Po)	Pistil	Pollen	288	200	30	589	431	3.10	57	97
P(L)	Pistil	Leaf	192	116	17	615	523	2.51	64	97
L(P)	Leaf	Pistil	192	60	34	576	447	2.15	74	98
Po(L)	Pollen	Leaf	192	129	28	543	500	2.40	70	92
L(Po)	Leaf	Pollen	192	158	16	565	525	2.52	77	95
Total			1344	790	171	579	478	2.64	70	95

*P, Pistil; Po, pollen; L, leaf*.

The P(Po) library provided information about those transcripts that are expressed during the pollen tube germination in comparison with the mature pollen grains, within the context of the whole pistil as in this stage, the pistil is full of germinating pollen grains. This library also offered information of the pistil transcripts. The P(L) library reveals the presence of transcripts in a tissue which is a distinct form from the leaf, being in addition a reproductive tissue. Lastly, the Po(L) shows the transcripts of a reproductive dormant tissue (pollen) from which transcripts from vegetative tissue have been subtracted.

A total of 1,344 clones were sequenced. From those, 790 resulted in ESTs and 171 in contigs. The mean length of the contigs compared to the ESTs showed an increase of 25% for Po(P), 27% for P(Po), 15% for P(L), 22% for L(P), 8% for Po(L), and 7% for L(Po). The redundancy levels were relatively low, ranging between 3.17 and 2.15%. BLAST analysis was carried out by using two alternative databases. The percentage of BLAST hits averaged 70% when the alignment was carried out with the NCBI database (http://www.ncbi.nlm.nih.gov; Altschul et al., 1990) (*e*-value e^−4^), and averaged 95% when the alignment was made against the OLEA EST database (Alagna et al., 2009) (*e*-values e^−1^).

In order to assess the subtractive efficiency of the libraries, PCR-amplified samples of the DNA inserts of each clone were transferred to membranes and subjected to multiple hybridizations with: (a) unsubtracted tester, (b) unsubtracted driver, (c) forward-subtracted, and (d) reverse-subtracted probes. An example of the procedure is displayed in Supplementary Figure 1.

The criteria followed in order to define clones as regard to their tissue-specificity were as follows: clones hybridizing exclusively with the forward-subtracted probe were considered to be differentially expressed. The clones that hybridize to the forward-subtracted probe and the unsubtracted tester probe also correspond to differentially expressed genes with a 95% of probability. Clones that hybridize to all the four probes correspond to non-differentially expressed clones. The results from the screening (Supplementary Tables 2–7) revealed that 33.3% of the clones were differentially expressed in the Po(P) library, 47.9% in the P(Po) library, 29.1% in the P(L) library, 41.6% in the L(P) library, 73% in the Po(L), and 68.2% in the L(Po).

**Figure 1:**
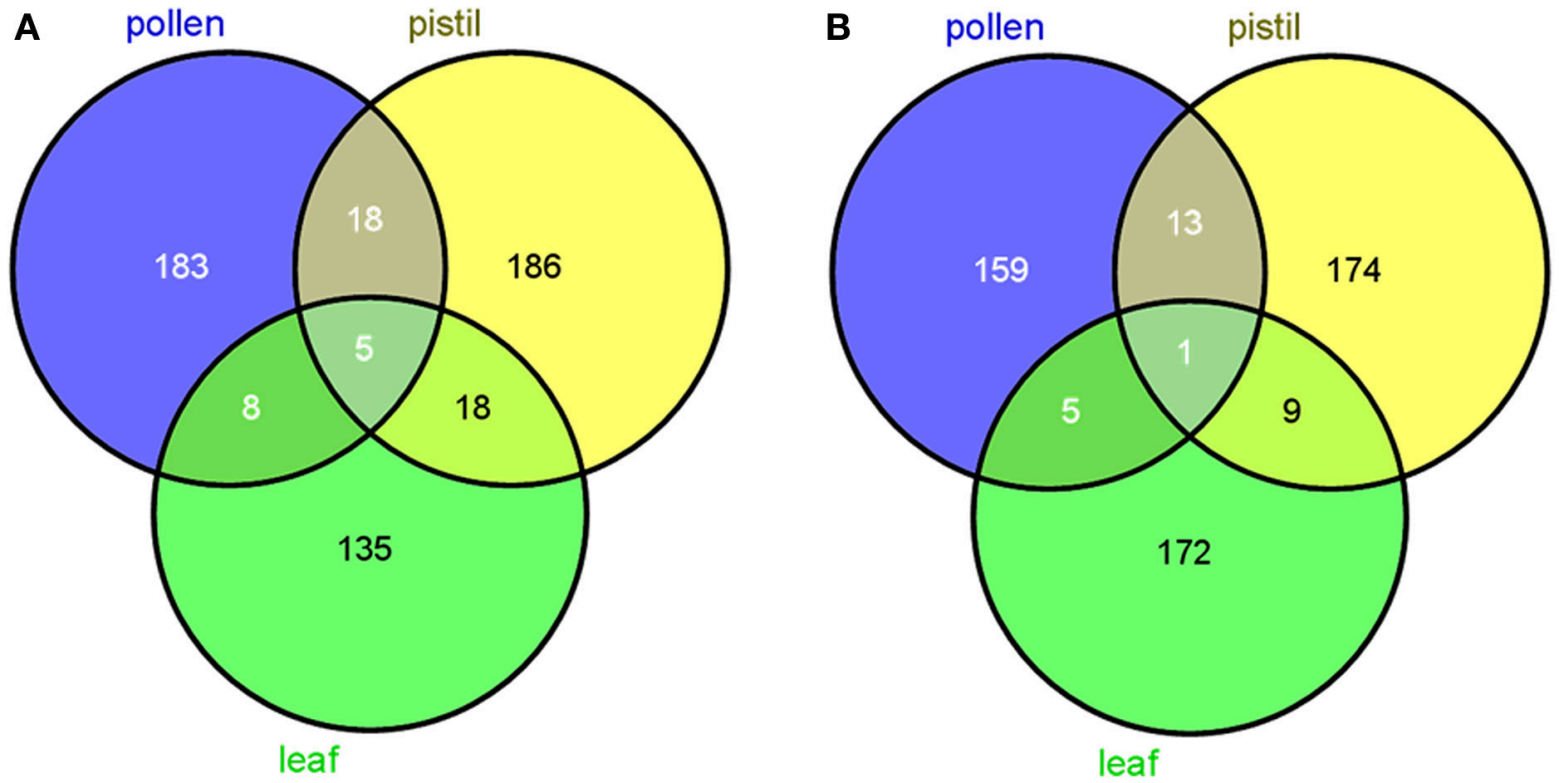
Venn diagrams showing the number of specific and common transcripts to the three tissues tested. **(A)** Output data after using OLEA ESTdb, and NCBIdb **(B)**, respectively (http://bioinfogp.cnb.csic.es/tools/venny/).

The use of subtractive libraries allowed us to obtain a relatively large number of tissue-specific transcripts. The number of such putative tissue-specific transcripts was highly dependent on the database searched by means of BLAST. Thus, the number of specific reproductive sequences (from pollen and pistil) was larger after using the OLEA EST db than after using NCBI db. The opposite tendency occurred with vegetative (leaf) transcripts (Figure 1).

**Figure 2:**
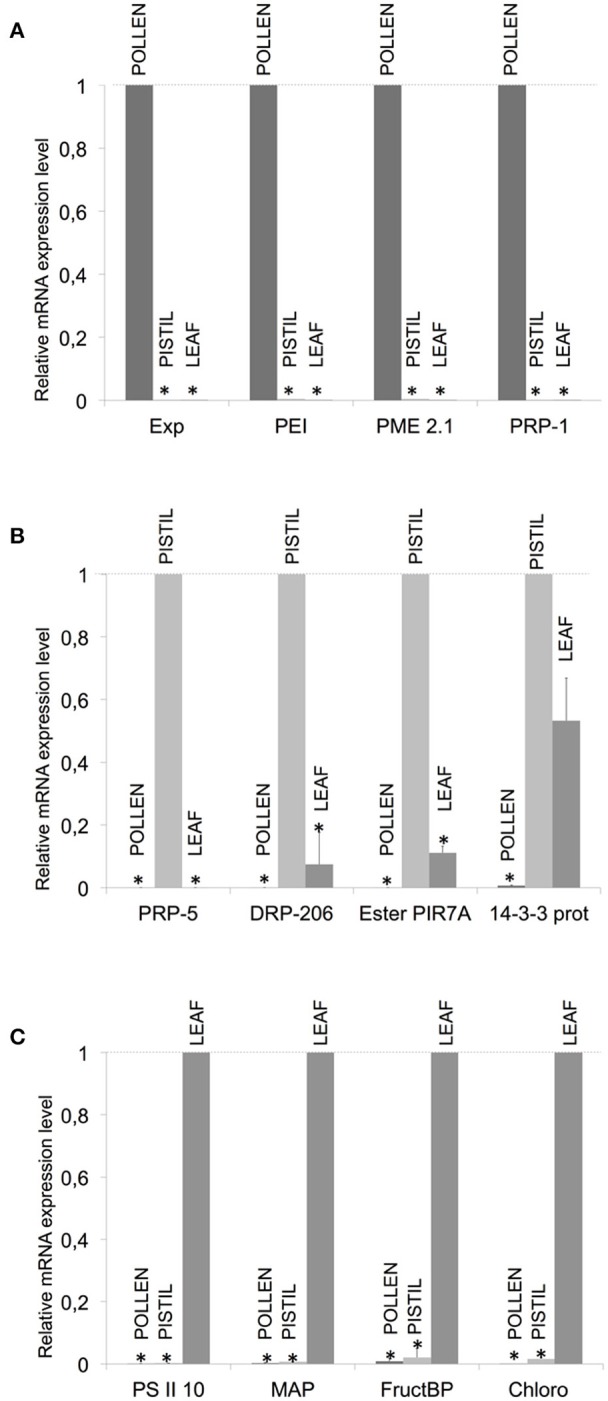
Validation of the generation of SSH libraries by qPCR of selected transcripts. Several pairs of primers were designed for pollen- (PoP library), pistil- (PPo library) and leaf-preferentially expressed (LPo library) transcripts (**A–C**, respectively). Exp, Expansin 11 precursor; PEI, Pectinesterase inhibitor 21; PME 2.1, Pectin metil esterase 2.1; PRP-1, Pathogenesis Related Protein-1; PRP-1, Pathogenesis Related Protein-5; DRP-206, Disease Resistance Response Prot-206; EsterPIR7A, Esterase PIR7A; 14-3-3 prot, 14-3-3 protein; PSII, Photosystem II 10 KDa polypeptide; MAP, Mitogen-activated protein kinase 3; FructBP, Fructose-bisphosphate aldolase; Chloro, Chlorophyll a-b binding protein. mRNA expression levels (average ± SD) of each transcript analyzed are shown in the three different tissues after normalization with the average expression of Katanin and Zinc-finger as housekeeping genes, made relative to the most expressed transcript (*y*-value: 1).

In order to validate the results obtained after the construction and screening of the SSH libraries, a total of 12 genes were selected for further assessment of their expression profiles by means of qPCR on the basis of representing a panel of different expression situations (Table 2). Four transcripts (Expansin 11 precursor, Pectinesterase inhibitor 21, Pectin methyl esterase 2.1 and Pathogenesis related protein-1) were selected according to their putatively unique or prevailing presence in pollen. The qPCR assays performed indicated that these transcripts were preferentially present in pollen (Figure 2A). Same approaches carried out with the remaining transcripts selected on the basis of their putative uniqueness or majoritarian expression in pistil and leaf (Table 2) yielded the qPCR results shown in Figures 2B,C, respectively.

**Table 2:** Selected gene products for pPCR validation.

**Name of the transcript**	**Sample**	**Po(P)**	**P(Po)**	**P(L)**	**L(P)**	**Po(L)**	**L(Po)**
Expansin 11 precursor	P1C2-PoP	+	−	−	−	−	−
Pectinesterase inhibitor 21	P2C45-PoP	+	−	+	−	+	−
Pectin metil esterase 2.1	P1C13-PoP	+	−	−	−	−	−
Pathogenesis related protein-1	P2C35-PoP	+	−	−	−	+	−
Pathogenesis related protein-5	P1C20-PPo	−	+	+	−	−	−
Disease resistance response protein-206	P1C40-PPo	−	+	−	−	−	−
Esterase PIR7A	P2C34-PPo	−	+	+	−	−	−
14-3-3 protein 4	P3C82-PPo	−	+	−	−	+	−
Photosystem II 10 kda polypeptide	P2C61-LPo	−	−	−	+	−	+
Mitogen-activated protein kinase 3	P1C13-LPo	−	−	−	−	−	+
Fructose-biphosphate aldolase	P2C28-LPo	−	−	−	+	−	+
Chlorophyll a-b binding protein	P2C39-LPo	−	+	−	+	−	+

Quantitative-PCR amplifications of all 12 transcripts showed the predicted patterns of tissue and abundance distribution predicted on the basis of their presence in the generated libraries. These 12 gene products exemplify a broad panel of functions and molecular characteristics, ranging from allergens, cell wall modification or loosening, secondary metabolism, hydrolase and catalytic activity, photosynthesis, oxidative stress, light reception and defense to pathogens. The importance of some of these functions in the reproductive process is discussed next.

**Figure 3:**
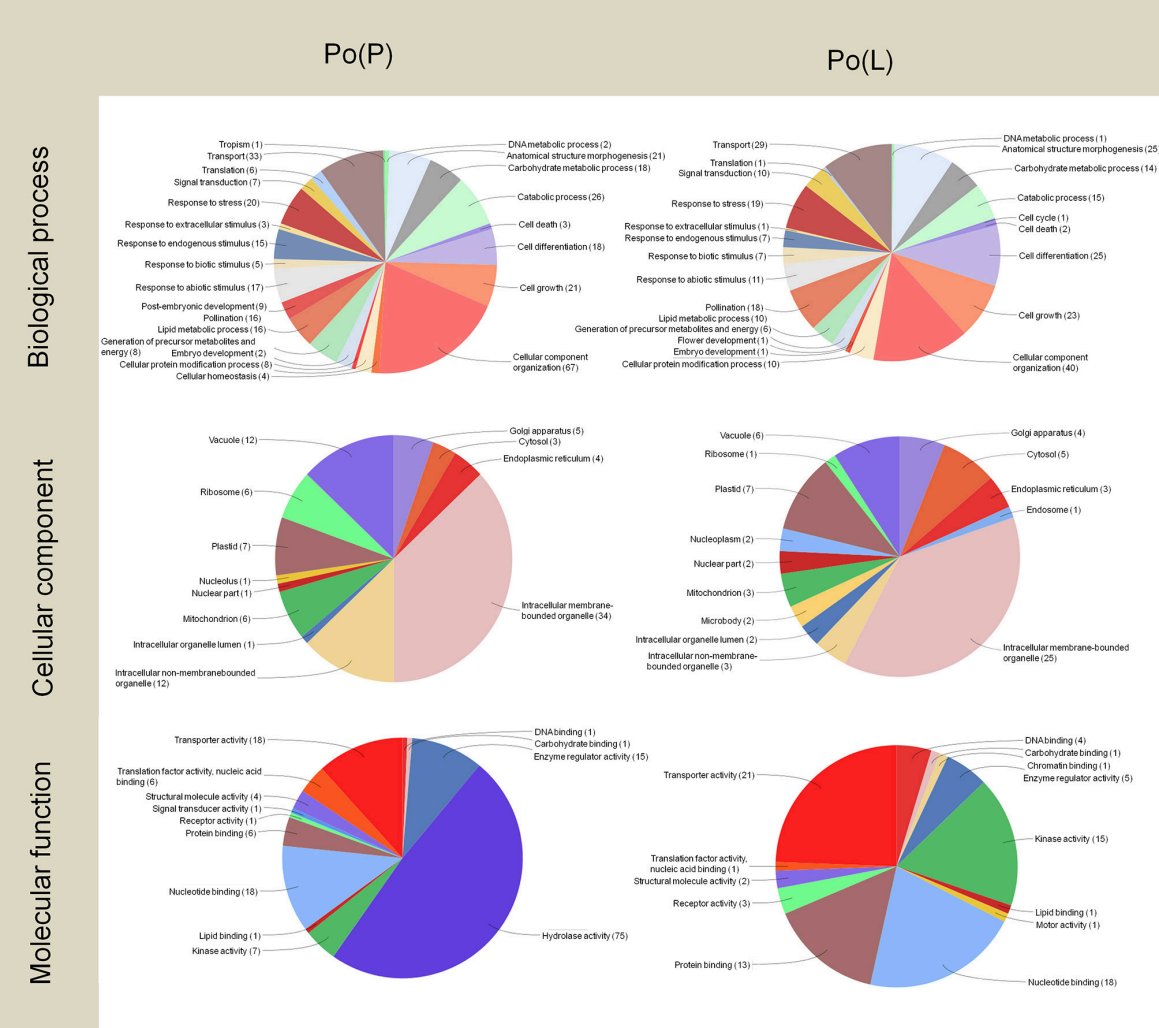
Gene Ontology terms distribution of the Po(P) and Po(L) libraries. The distribution of biological processes and molecular function was assessed to a sequence cut off = 1. The distribution of cellular components corresponds to a level = 6. The graphs were prepared using the Blast2Go software.

**Figure 4:**
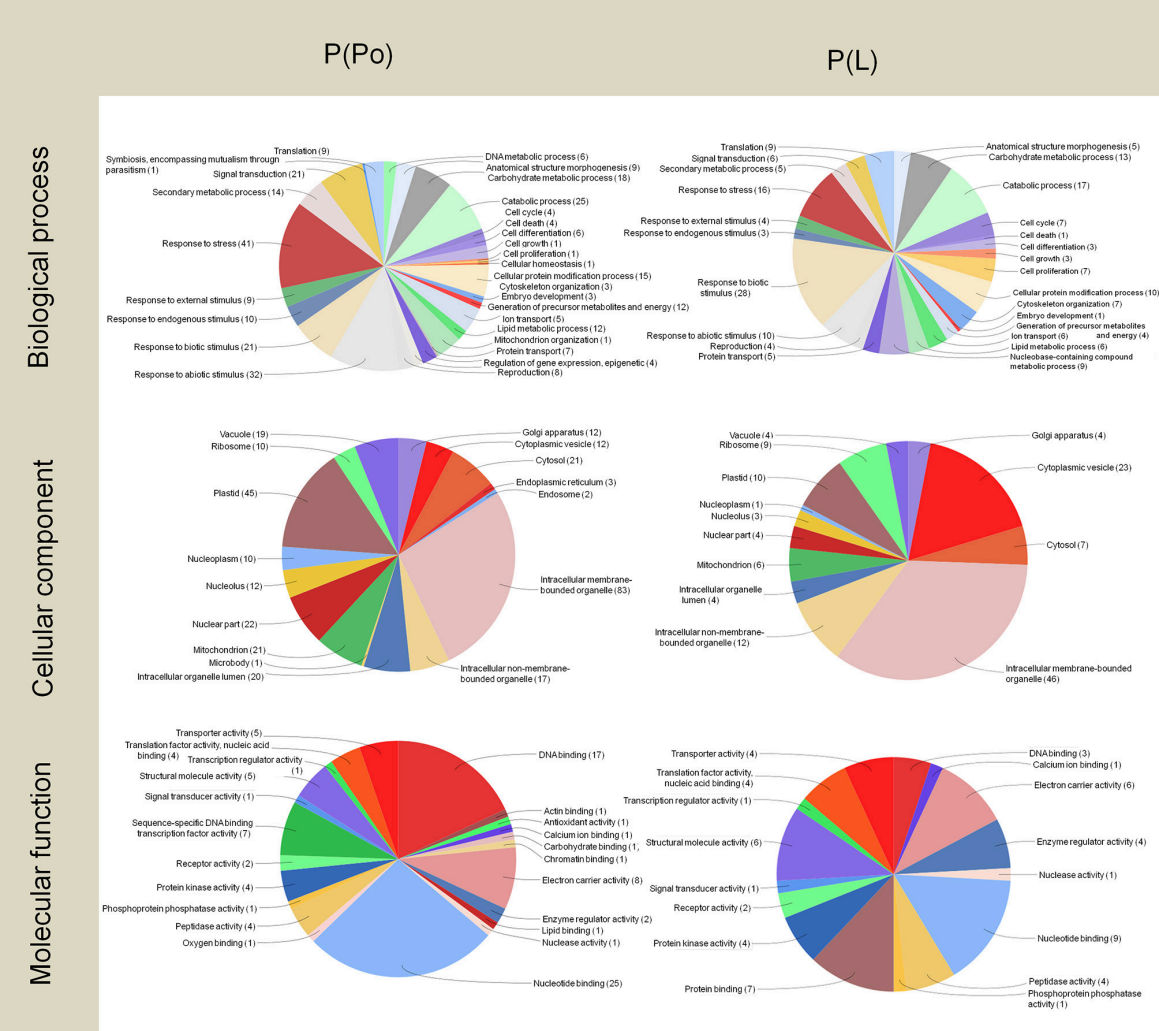
Gene Ontology terms distribution of the P(Po) and P(L) libraries. The distribution of biological processes and molecular function was assessed to a sequence cut off = 1. The distribution of cellular components corresponds to a level = 6. The graphs were prepared using the Blast2Go software.

**Figure 5:**
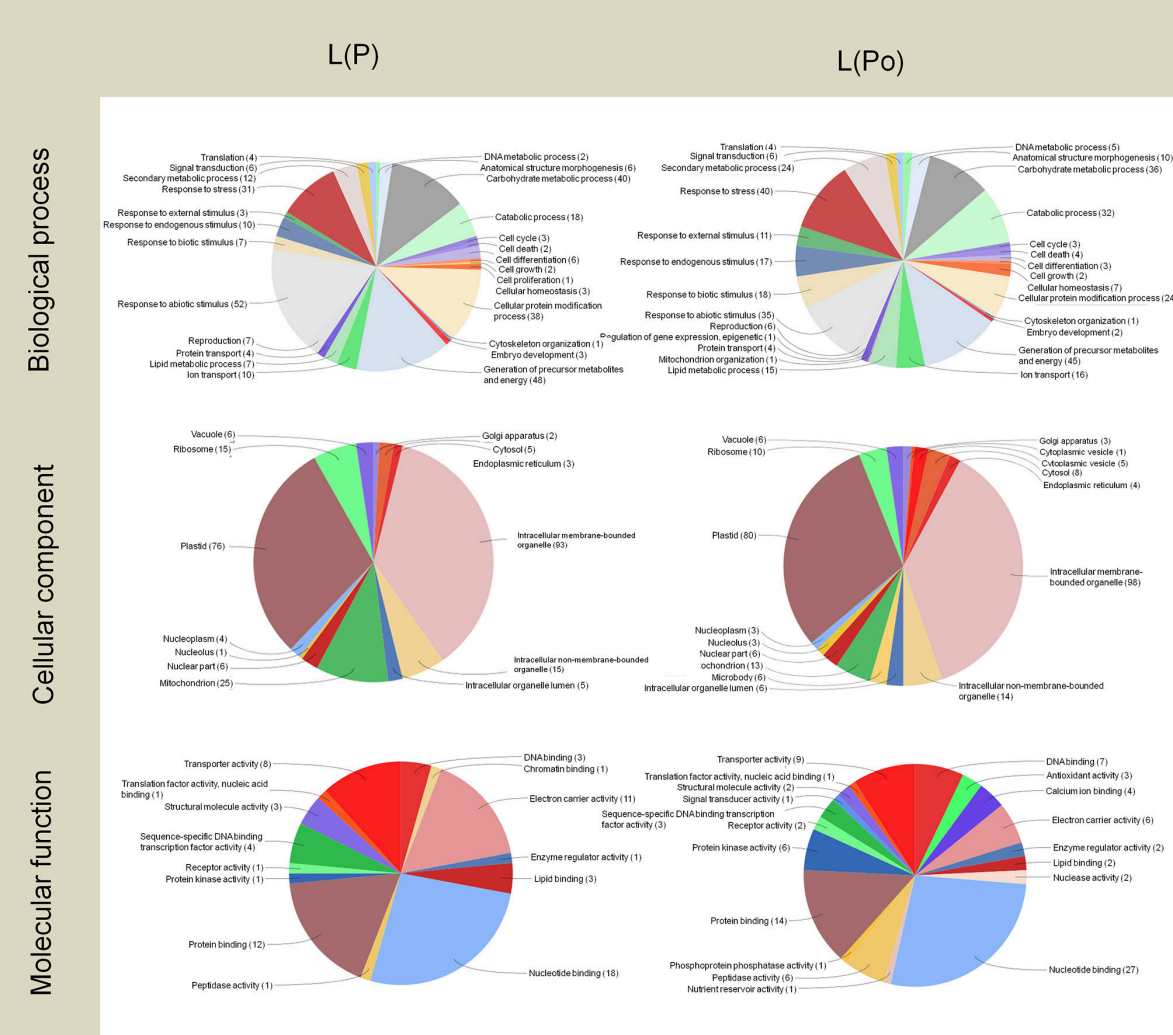
Gene Ontology terms distribution of the L(P) and L(Po) libraries. The distribution of biological processes and molecular function was assessed to a sequence cut off = 1. The distribution of cellular components corresponds to a level = 6. The graphs were prepared using the Blast2Go software.

The concentration of high- and low-abundance sequences was equalized in the different libraries, which allowed us to identify low abundance transcripts but with the drawback of missing details about their real abundance. However, analysis of the distribution of Gene Ontology terms provided a first approach to define the implication of these transcripts (Figures 3–**5**). The two pollen subtractive libraries [Po(L) and Po(P)] (Figure 3) included exclusive transcripts involved in biological processes related to the categories of pollination, responses to extracellular stimulus and post-embryonic development, and a high abundance of transcripts connected with cell differentiation, cell growth, and cellular component organization, in comparison to the rest of the libraries. The large presence of transcripts involved in cellular component organization may suggest that although the mature pollen grains have not yet started to germinate, many transcripts needed for pollen tube formation are already accumulated inside the pollen grains. In the same way, transcripts connected to transport activity also occupy a relevant role in both pollen libraries [Po(P) and Po(L)]. Therefore, the presence of such “preparative” transcripts in the mature pollen grain may be a determinant for the correct development of the pollen tube once the pollen grain arrives on the stigma and starts germinating through the style.

As regard to the distribution of transcripts among the different cellular components, the presence of transcripts apparently targeted to plastids is surprising, as olive pollen grains contain only poorly-differentiated plastids, probably lacking a highly structured biochemical machinery (Rodríguez-García and García, 1978; Rodríguez-García et al., 1995). In both the Po(P) and Po(L) libraries, transcripts likely assigned to intracellular membrane-bounded organelle represent approximately one-third of the total transcripts.

Regarding the pie charts showing molecular function, considerable differences between the two pollen subtractive libraries were observed. These differences mainly result from the massive presence of Pectin Methyl Esterases (PMEs) in pollen. PMEs are enzymes present in higher plants, fungi and bacteria. They catalyze the demethylesterification of homogalacturonan residues of pectin, releasing methanol as the reaction product. Such modification is responsible for changes in the pectin molecule, which can then be cross-linked by calcium, and this further results in changes in the mechanical properties of the plant cell wall, altering its plasticity. This particularly affects the ability for growth and guidance of pollen tubes (Castro et al., 2013). Pollen specific PMEs have been described in other species (Tian et al., 2006; Gómez et al., 2013), with key roles during pollen germination (Leroux et al., 2015), during pollen tube elongation along the transmitting tract and when the pollen tube reaches the embryo sac in the ovule (Gómez et al., 2013). The olive pollen PME is considered a highly prevalent allergen present in the olive pollen (Salamanca et al., 2010).

Approximately half of the transcripts in the Po(P) library were involved in hydrolase activity. On the other hand, the Po(L) library did not show a high abundance of these transcripts. The subtraction carried out to create the Po(P) library possibly removed most of the PMEs of the pollen, and allowing the identification a wide variety of PME isoforms, as well as one pollen-specific PME. Therefore, the information provided by the pie charts for molecular functions delivers particular evidence on the processes happening within the pistil at stage 4.

In the pistil subtractive libraries P(Po) and P(L) (Figure 4), the presence of an exclusive transcript related to symbiosis (encompassing mutualism through parasitism) was detected. Further to this, both the pistil and the leaf tissues contain wide pools of transcripts related to stress and defense. A highlight here is the presence of transcripts linked to responses to biotic stimulus in the P(L) library as it has been proposed that certain self-incompatibility processes may have evolved from pathogen-defense mechanisms (Hodgkin et al., 1988; de Nettancourt, 1997; Elleman and Dickinson, 1999; Hiscock and Allen, 2008). Within these transcripts, we found Pathogenesis related proteins-1, 5, and 10, Beta-glucosidases and PME Inhibitors.

On the other hand, the pistil harbored numerous PME inhibitor transcripts, but they did not appear in the P(Po) library as the consequence of the subtraction carried out. This is likely due to the homology between PME and the PME inhibitors (PMEI) in the N-terminal pro-region, present in the stigmatic exudate of the olive (Rejón et al., 2013). This similarity among many plant species was previously described by Jolie et al. (2010) suggesting the inhibitory role of the PME pro-peptide region. PMEI may be originated from a rearrangement of plant genome which could have the starting point of the PME inhibitors (Giovane et al., 2004) dare consistent with the localization of the PME inhibitors described at the pollen tube apex (Röckel et al., 2008), which were detected in the pistil library, probably as a result of the presence of pollen tubes growing through the stigma-style.

In a similar way to both pollen libraries, there was a large presence of transcripts for proteins associated to intracellular membrane bounded organelles in the P(Po) and P(L) libraries. Among these, transcripts associated to plastids scored as an important proportion, and were more numerous in the P(Po) library than in the P(L) one.

The analysis of the molecular functions of the transcripts present in pistils revealed the noticeable presence of transcripts for proteins with electron carrier activity in both cases, which did not appeared in the pollen libraries. The number of transcripts for proteins with DNA-binding was almost six-fold higher in the P(Po) library compared to the P(L) library. Even though transcripts within the nucleotide-binding category were abundant in both libraries, they were almost three times more abundant in the P(Po) library than in the P(L) one.

DNA-binding proteins are key players in the process of expression and regulation of new proteins as such interactions are considered to be central for many basic biological processes, including transcription regulation, DNA replication and DNA repair (Bonocora and Wade, 2015). The high levels of transcripts encoding DNA-binding proteins in the pistil could be indicative of the ability to have quick responses, where finely tuned regulation is needed. On the other hand, the presence of transcripts for nucleotide binding proteins could also be related to the changes happening outside the gynoecium cells, as the plant disease resistance genes have been described to frequently encode nucleotide binding proteins (Meyers et al., 1999).

Regarding the leaf subtractive libraries L(Po) and L(P) (Figure 5), most of the transcripts corresponded, not surprisingly, to proteins involved in photosynthetic metabolism, with the detection of a large proportion of transcripts for proteins located at plastids as well as a majority of transcripts implicated in biological processes such as carbohydrate metabolism and the generation of precursor metabolites and energy. This is also consistent with the large presence of transcripts for proteins with electron carrier function. As expected, these transcripts are also present in the pistil, although less abundantly, and absent in pollen.

To sum up and as discussed above, the putative origins, biological processes, cellular localizations and molecular functions of the transcripts identified in the different subtractive libraries analyzed are in good agreement with the predicted nature of such transcripts derived from the methods and tissues used for their construction, as displayed in Table 3. It is necessary to take into account that pistil tissues are expected to include dehiscent (mature) olive pollen, as well as hydrated pollen grains and even germinating pollen grains and pollen tubes, as described in the methods section.

**Table 3:** Putative composition and nature of the transcripts present in the generated SSH libraries.

**Name of the library**	**Tester tissue**	**Driver tissue**	**Expected biological significance**
Po(P)	Pollen	Pistil	Transcripts expressed exclusively in mature pollen grains, not expressed in pistil nor in hydrated, or germinated pollen grains.
P(Po)	Pistil	Pollen	Transcripts expressed exclusively in pistil and hydrated and/or germinated pollen grains but not expressed in mature pollen grains.
P(L)	Pistil	Leaf	Transcripts expressed exclusively in pistils, but not in vegetative tissues (leaf) despite their common evaluative origin.
L(P)	Leaf	Pistil	Transcripts expressed exclusively in vegetative tissues (leaf) and hydrated or germinated pollen grains, but not in pistil.
Po(L)	Pollen	Leaf	Transcripts expressed exclusively in male reproductive tissues (pollen grains), but not in vegetative tissues (leaf).
L(Po)	Leaf	Pollen	Transcripts expressed exclusively in vegetative tissues (leaf), but not in mature pollen grains.

**Figure 6:**
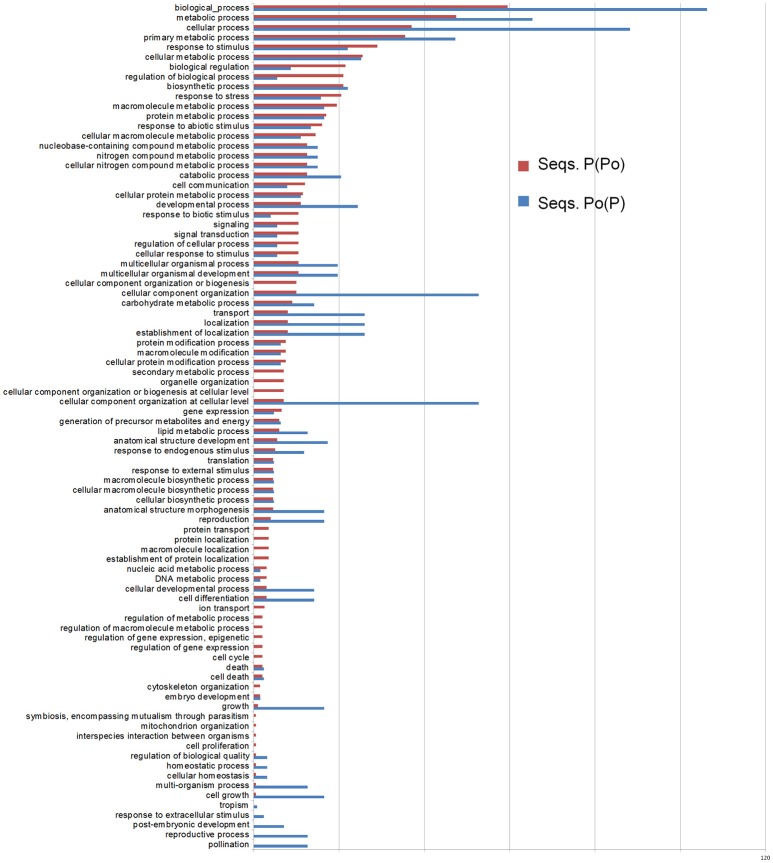
Differential Gene Ontology terms between the Po(P) and P(Po) libraries. The distribution of GO terms has been created as relative percentage considering the number of total ESTs obtained for each library.

Therefore, taking into account the origins for the described transcripts, the SSH libraries which best describe the pollen-pistil interactions in olive and the pollen hydration and pollen tube growth are Po(P) and P(Po). For both of these SSH libraries, the corresponding GO terms have been identified, and been represented together for comparison purposes (Figure 6).

Within the pool of transcripts present in the pistil from which pollen has been subtracted P(Po)], those involved in regulation, response to stress/stimulus, and signaling/cell communication are more abundantly represented in terms of relative percentage. On the other hand, the library of pollen from which pistil has been subtracted [Po(P)], is mainly rich in transcripts involved in cellular organization, localization, developmental processes, pollination and growth. Detailed lists of the transcripts detected for each pollen SSH library [Po(P) and Po(L)], together with BLAST relevant scores for each one are listed in Supplementary Tables 2, 6. Amongst these pollen transcripts it was found LAT52, which is known to play a role in pollen hydration and germination (Muschietti et al., 1998; Tang et al., 2002). The presence of SF21, with a putative function in pollen-pistil interaction (Allen et al., 2010a) confirms the importance of these transcripts in reproduction. However, no transcripts of SF21 were found in the pistil [see Supplementary Tables 3, 4, corresponding to P(Po) and P(L)], despite a putative function in pollen tube guidance (Allen et al., 2010a). The Soluble NSF Attachment Protein Receptor (SNARE) proteins within the mature pollen grains are also present in the spore, with a role in the pollen tube movements (Bushart and Roux, 2007).

In the pistil (Supplementary Tables 3, 4), the response to stress is mainly represented by the Pathogenesis related proteins (PRPs); the signaling processes occurring in the pistil is emphasized by the presence of auxin responsive factors. Regulation is carried out by the interaction with the pollen specific auxin induced/repressed proteins (present in the mature pollen; they were found in both pollen libraries). The output results from the pistil libraries showed a similarity to that seen with auxin-induced root cultures (Neuteboom et al., 1999), but with an unknown function in the mature pollen grains. The auxin responsive proteins present in both pistil libraries are important for pollen tube formation (Yang et al., 2013), which indicates the presence of growing pollen tubes. However, they are not present in the mature pollen grains (Supplementary Tables 2, 6). The 14-3-3 protein is also important in pollen germination as it is involved in the regulation of turgor pressure of the pollen tube (Pertl et al., 2010).

The use of BLAST analysis of the SSH-retrieved sequences against the OLEA ESTdb, specifically containing sequences from olive mesocarp only, provided in many cases further information through the annotation of our sequences. For example, the described pistil-specific thaumatin/PRP-5 (Kuboyama, 1998; Sassa et al., 2002) is identified in the OLEA ESTdb as “Thaumatin-like protein, Pathogenesis–related protein 5,” whereas the NCBIdb identify them with the general term “Thaumatin-like protein.” We were able to discriminate between two different thaumatin-like proteins: Pathogenesis–related protein 5, which was only found in the pistil, and the Pathogenesis–related protein 1, which was pollen specific. Moreover, three isolated transcripts from different thaumatins were found in the pistil, the first one identified as “STS14 protein/ Pathogenesis-related protein 1C,” and the others as “Osmotin-like protein” (OSML13 and OSM34, respectively). The STS14 protein is proposed to be involved in the protection of the outer tissues of the pistil from pathogen attack or guidance of the pollen tubes through the pistil. It is highly expressed in the stigma and stylar cortex around 120 h before anthesis and increases toward the end of flower development, with a maximum at anthesis (Van Eldik et al., 1996).

**Figure 7:**
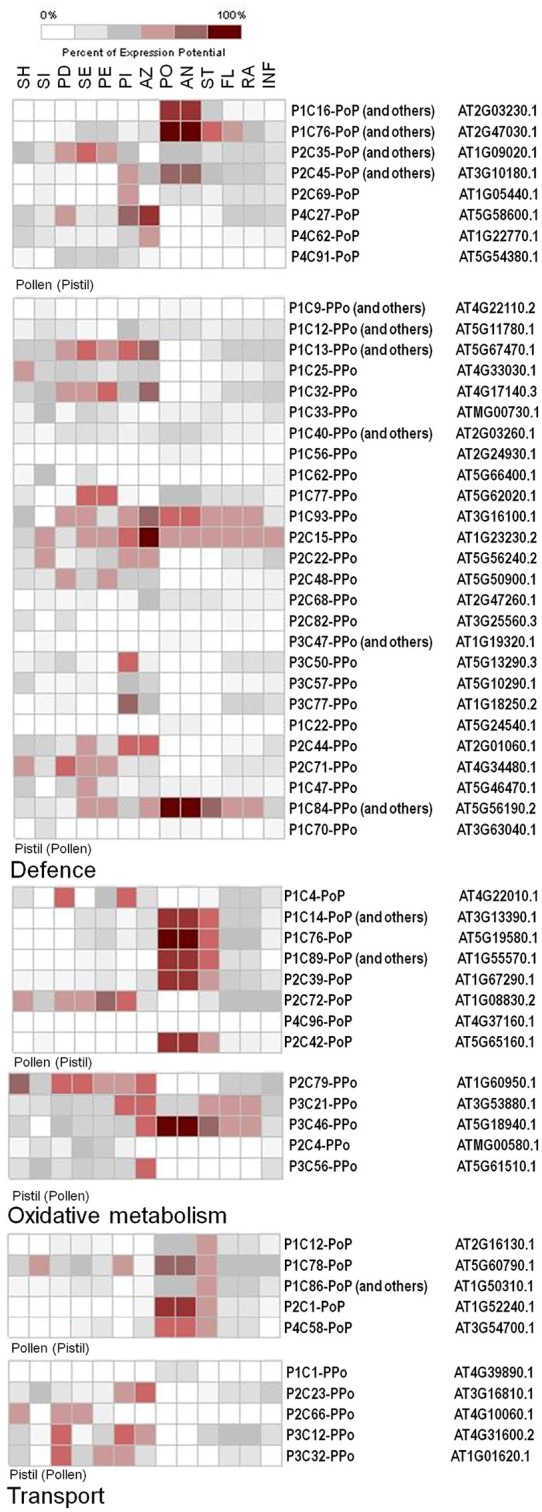
Level of expression across several reproductive/vegetative tissues of genes considered putative homologs to *Arabidopsis* using the anatomy tool Genvestigator. Three categories were considered for analysis of the levels of expression: oxidative metabolism (upper part), defense (middle part), and transport (lower part). The transcripts of two selected libraries were considered only: Po(P) and P(Po). SH, shoot; SI, silique; PD, pedicel; SE, sepal; PE, petal; PI, pistil; AZ, abcision zone; PO, pollen; AN, anther; ST, stamen; FL, flower; RA, raceme; INF, inflorescence. Identities of both the olive SSH transcripts and their corresponding Arabidospsis homologous are shown for reference purposes.

As an example, three groups of transcripts were selected (defense, oxidative metabolism and transport: Figure 7) with the aim to further analyze and discuss the expression of transcripts with high abundance as well as their key putative roles in the pollen-pistil interaction, pollen tube germination and growth. The genes considered putative homologs to *Arabidopsis* from each group were analyzed throughout the anatomy tool of Genvestigator. The specificity of the transcripts and the biological implications of their differential expression are described and discussed below.

### Different transcripts putatively involved in defense are present in the pistil

One of the stigma-specific transcripts detected in olive was that corresponding to the Pathogenesis-related protein 5 (PRP-5). Members of this protein group have been associated with resistance to fungal infection and to responses to biotic/abiotic stresses, disease resistance or hormonal responses by inducing transcripts such as DOR, MYB, AP2, and WRKY (El-kereamy et al., 2011). A pistil-specific thaumatin/PRP-5 has been described in Japanese pear (Sassa et al., 2002) and in tobacco (Kuboyama, 1998), where the maximum levels of the transcript were reached at anthesis. This gene product has been proposed to play a role in pollen recognition and pollen tube pathways. Among the stigma-specific PRP-5 sequences obtained in olive, we observed high homology with the SE39B specific-stigma thaumatin from tobacco (Kuboyama, 1998) and also with a specific thaumatin from the fruit of *Olea europaea* highly expressed in response to phytophagous larvae (Corrado et al., 2012). Another olive stigma-specific transcript of interest, involved in defense, and also considered an allergen, is Mal d 1 from apple (Vanek-krebitz et al., 1995). This belongs to the PRP-10 group. These gene products have been described throughout several developmental stages and plant tissues, with a dual role associated with defense functions and regulation/signaling (Zubini et al., 2009; Choi et al., 2012). Pectin Methyl Esterases (PMEs) and their inhibitors (PMEIs) have been considered to be involved in defense function in vegetative tissues (McMillan et al., 1993; Boudart et al., 1998; Lionetti et al., 2007, 2012; Wydra and Beri, 2007; Ann et al., 2008; Körner et al., 2009; Volpi et al., 2011, 2013). Thus, the PMEs have been reported to enhance RNAi action, acting in gene-regulatory mechanisms (Dorokhov et al., 2006), which include virus-induced gene silencing (VIGS) and the fight against other pathogens (Collmer and Keen, 1986). Interestingly, it has been suggested that PMEIs might be internalized by endocytosis at the flanks of the pollen tube tip, regulating pollen-tube wall stability by locally inhibiting pollen PME activity (Röckel et al., 2008). It has also been suggested that PMEIs are able to reduce the activity of cell wall PMEs, leading to a drop-in pollen tube stability (Paynel et al., 2014). A pollen specific PMEI was described in broccoli triggering partial male sterility and decreased seed set by inhibition of pollen tube growth (Zhang et al., 2010).

The large presence of cysteine proteinase in the pistil may be attributed to defense mechanisms, similar to that already described by Grudkowska and Zagdańska (2004). Another defense mechanism which seems to work actively in the olive pistil is the “disease resistance response protein 206,” that has been described to be induced in pea in response to the infection by *F. solani* f. sp. *phaseoli* (Culley et al., 1995). Within the Late Embryogenesis Abundant proteins, the pistil possesses transcripts for the dehidrin Rab 18. The Late Embryogenesis Abundant-18 transcript decreases during the germination process in pea, though it is present again in the emerging hypocotyls. Therefore, this transcript might be related to the elongation process under optimal growing conditions (Colmenero-Flores et al., 1999). In the case of the olive pistil, the presence of these transcripts could be related to the elongation process occurring in the growing pollen tubes within the stigma/style. Other transcripts found in the pistil libraries were: beta-glucosidases, late blight resistance proteins, WRKY genes, mitogen-activated kinase proteins, and the MYB genes expressed in the olive pistil are also involved in defense (Pandey and Somssich, 2009; Engelhardt et al., 2012). The MYB transcription factor itself has been described to be involved in pollen development (Niwa et al., 1993; Katiyar et al., 2012). A pistil specific nodulin has been also described in the pistil of several species (Allen et al., 2010b), being involved in a successful fertilization (Shi et al., 2012).

### Different transcripts putatively involved in defense are present in the pollen grain

Defense genes highly expressed in the olive pollen also comprise PME again, the PME inhibitor U1, and a panel of eight pathogenesis-related proteins.

PRP-1 was detected exclusively in olive pollen subtracted Po(P) and Po(L) libraries. To date, PRP-1 has only been described to be involved in food allergy (Asensio et al., 2004), as the precise function of these proteins is not in the pollen itself known. The specific expression of the heat stress transcription factor (HsfA2) was also detected. HsfA2, together with chaperones, are important protectors of the pollen maturation, viability and pollen tube germination from heat damage (Frank et al., 2009; Giorno et al., 2010; Zinn et al., 2010).

### Oxidative metabolism in the pistil

Closely related to defense mechanisms, oxidative metabolism interplays a dual role, keeping the balance between defense and signaling. In the case of the pistil, these two functions are even more finely tuned as the signaling processes are very important for a successful reproduction. Therefore, it is important to highlight the presence of transcripts corresponding to Glutathione S-transferases (GSTs), Ferredoxin-1, NAD(P)H-dependent oxidoreductase, Peroxidase 72 and Quinone oxidoreductases. Most of these transcripts have not been described as pistil-specific in Arabidopsis (Figure 7). Among these, stigma-specific peroxidases have been previously studied in several species (McInnis et al., 2005; Swanson et al., 2005; Beltramo et al., 2012), with the implication in the pollen-pistil interaction, pollination process, and signaling. The glutathione S-transferase has been classified as an allergenic protein in animal species (Yu and Huang, 2000; Huang et al., 2006). Later it was identified in birch pollen (Deifl et al., 2014). However, when compared to other birch pollen allergens such as Bet v 1, the release kinetics of Glutathione S-transferase from pollen grains upon contact with water and different physiologic solutions was much slower. It was suggested that the amount of glutathione S-transferases released during this time period was too low to induce allergic sensitization (Deifl et al., 2014).

### Oxidative metabolism in the pollen grain

The presence of transcripts from Tpr repeat-containing thioredoxin ttl1-like was observed. Such gene products have been described to accumulate in response to osmotic stress and abscisic acid (ABA), and also may be involved in pollen compatibility (Haffani et al., 2004). Using analysis to look for members of the oxidoreductase family of proteins we could find transcripts for galactose oxidase, glyoxal oxidase and a specific L-ascorbate oxidase homolog (Pollen-specific protein NTP303). To our knowledge, the presence of galactose oxidase has not been connected to any particular characteristic of the plant reproductive tissues. Interestingly, the enzyme glyoxal oxidase has been described to be involved in male sterility, jointly to other enzymes implicated in cell wall expansion (Chen et al., 2012; Suzuki et al., 2013). The presence of L-ascorbate oxidase transcripts has been described in *in vitro* germinating pollen grains (Weterings et al., 1992), although we failed to find these mRNAs in the olive pistil, which also contains *in vivo* growing pollen tubes. It is interesting to highlight the presence of the olive pollen allergenic protein Cu, Zn Superoxide Dismutase which is involved in the protection against oxidative stress during pollen development. Its dual role, i.e., as an allergen and as part of the antioxidant/signaling metabolism, makes its study particularly interesting (Butteroni et al., 2005). Moreover, it has been described to be implicated in the development of the male reproductive tissues of the olive tree (Zafra et al., 2012).

### Transcripts connected with transport of molecules in the pistil

Pollen-stigma interactions and the growth of the pollen tube throughout the pistil tissues encompass a large exchange of molecules among these tissues, either positively or negatively regulating and/or permitting such growth, throughout providing energy, ions or structural molecules. Among the pistil preferential transcripts detected in this work, several have been attributed with functions facilitating transport of such molecules. This is the case for the Ras-related transport protein, which facilitates proteins movement through membranes, and the mitochondrial import inner membrane translocase subunit Tim13. Transcripts from a member of the solute carrier family 35 (B1) are also present in the olive gynoecium. Other transporters that have been described also in primary roots (with a growing processes comparable to that of pollen tubes within the style of receptive flowers) are the specific lipid-transfer protein (LTP) AKCS9 (present in membranes) and aquaporins, both specifically present within the olive pistil transcripts and with described vegetative/reproductive differential meanings: lipid-transfer proteins were correlated with root hair deformation and pistil abortion (Krause et al., 1994; Shi et al., 2012) whereas specific aquaporins were found in the region adjacent to the root tip and have been demonstrated to be required for the self-incompatibility process displayed for members of the family *Cruciferae* (Ikeda et al., 1997; Sakurai et al., 2008).

### Transcripts connected with transport of molecules in the pollen grain

Transcripts for several transporters were found in the mature olive pollen grain. The sugar transport protein must represent a key transcript in pollen and pollen germination as it has been described in tobacco (Lemoine et al., 1999). Also, the polyol transporter present in the olive pollen could share similar functions to the polyol/monosaccharide transporter 2 expressed in mature pollen grains, growing pollen tubes, hydathodes, and young xylem cells (Klepek et al., 2010). Moreover, boron transporters expression reveals the regulatory role of boron in pollen germination and pollen tube growth (Qinli et al., 2003). Nitrate transporters also act as a nitrate sensor that triggers a specific signaling pathway stimulating lateral root growth (Guo et al., 2001), which may have a similar significance in pollen tube growth. The presence of the cation proton exchanger is critical for maintaining polarity, directing pollen growth toward the ovule, and to allow cell expansion and flower development (Bassil et al., 2011; Lu et al., 2011). The transcripts encoding ABC transporters, also found in the olive pollen, could be related to the transport of sporopollenin precursors for exine formation in developing pollen (Choi et al., 2011). Rho guanine nucleotide exchange factors are crucial in polar growth of pollen tubes (Zhang and McCormick, 2007). Finally, phosphate transporters have also been described as central for gametophyte development (Niewiadomski et al., 2005).

### Other transcripts

The present analysis also has reported some unexpected results. As an example, anther-specific proline-rich protein APG transcripts have been found in the pistil, when they have been considered to be confined to the anther during the period of microspore development, with a dramatic decline during pollen maturation (Roberts et al., 1993). This result could be explained by the implication of the proline-rich protein APG in the pollen tube during the germination process, through a process yet to be determined.

Even though our data still do not reveal substantial information as regards some key aspects of the olive reproductive biology which are still open, such as the demonstration of the presence of a self-incompatibility system of the gametophytic type (largely suspected). Many of the transcripts detected here (either tissue-specific or not) are of great interest for the further characterization of the species, and in some cases for important issues like olive pollen and stigma physiology, as discussed above. Current knowledge of olive pollen allergenicity can also be improved, as several of the identified transcripts correspond to potential allergenic molecules already described in other species, but as such not yet described in olive. This is the case, for example, with glutathione S-transferase, considered a minor allergen in birch pollen (Zwicker, 2013; Deifl et al., 2014). Gene products corresponding to transcripts detected in the resulting SSH libraries P(Po) and P(L) described here are also consistent with proteins characterized in the olive stigma exudate by means of proteomic approaches (Rejón et al., 2014), which may act as Additional positive controls for the present methodology, because the presence of olive pollen originated peptides among those detected by was almost completely avoided.

## Comparative transcriptomics

Comparative analysis of the SSH-derived transcripts with transcriptomic information present in two recently developed databases (ReprOlive: Carmona et al., 2015; Cruz et al., 2016) was preceded by a bioinformatics cleaning by SeqTrimNext which resulted in 820 useful sequences retained from 1,344 clones sequenced with the distribution displayed in Table 4.

**Table 4:** Summary stats about the pre-processing of the libraries.

**Name of the library**	**Number of sequences before cleaning**	**Number of sequences after SeqTrimNext treatment**
Po(P)	288	191
P(Po)	288	137
P(L)	192	119
L(P)	192	91
Po(L)	192	141
L(Po)	192	141
Total	1344	820

Further annotation of the cleaned sequences from pollen libraries [Po(P) and Po(L)] and screening for correspondence with the two transcriptomic databases yielded the information displayed in Supplementary Tables 8, 9. Similarly, Supplementary Tables 10–13 include the correspondence of pistil libraries [P(Po) and P(L)] and leaf libraries [L(Po) and L(P)] with both transcriptomic databases.

Overall, a high proportion (88.5–100%, with an average of 95.8%) of the SSH sequences identified here mapped to both transcriptome databases (Table 5). Tissue specificity of the SSH sequences described here, assessed by BLAST between the different ReprOlive datasets with Roche/454 reads (Additional files 8–13), also yielded a high proportion of “YES” matches at the appropriate tissue, thus validating *in silico* the usefulness of the SSH approach which was carried out experimentally here.

**Table 5:** Percentages of “pre-cleaned” SSH transcripts mapping to the two recently developed olive transcriptome databases (ReprOlive: Carmona et al., 2015; Cruz et al., 2016).

**Name of the library**	**Number (%) of sequences mapping to ReprOlive**	**% of sequences mapping to the olive genome-derived transcriptome**	**Average (%)**
Po(P)	189 (99)	169 (88.5)	93.8
P(Po)	135 (98.5)	127 (92.7)	95.6
P(L)	115 (96.6)	110 (92.4)	94.5
L(P)	91 (100)	89 (97.8)	98.9
Po(L)	140 (99.3)	129 (91.5)	95.4
L(Po)	136 (96.5)	136 (96.5)	96.5
Average	98.3	93.2	95.8

## Conclusions

The generation and analysis of different SSH subtractive libraries has provided a dataset of sequences, consisting in about a thousand entries of great value for the understanding of the physiological processes taking place in olive pollen and pistil during their development and interaction. They are particularly important as many of these inputs have been demonstrated to be exclusively or preferentially expressed in the reproductive tissues, and not in the leaf tissues, as this material was used to build the subtractive strategy.

The subtractive transcripts have been annotated according to their homology as regard to four main databases: a general plant database provided by the NCBI, and three olive-specific databases constructed from mesocarp, reproductive tissues and a final one derived from the lately published olive genome draft. Moreover, they have been extensively classified and their presence discussed as regard to their putative biological function, cellular localization, and the molecular functions expected to exert.

Such information will be used in the near future as the basis to examine further aspects of the olive reproductive biology through the specific analysis of the expression of these products. These aspects may include compatibility, cell-to-cell communication, pollen tube growth and guidance, and pollen allergenicity among others.

## Author contributions

AZ and JA designed the experiments and redacted the manuscript. AZ performed the experiments and analyzed the results. JT was particularly involved in the work with the databases and tools on the web servers. SH and JH took part in the lab hosting and supervision during the AZ stay in their respective laboratories. MHG participated in the sequencing and interpretation of results. RC and MGC performed database searches and other bioinformatics analyses and organized and deposited all sequences at ENA.

### Conflict of interest statement

The authors declare that the research was conducted in the absence of any commercial or financial relationships that could be construed as a potential conflict of interest.
