# Supplementary figures and images for: Identification and Functional Annotation of Genes Differentially Expressed in the Reproductive Tissues of the Olive Tree (Olea europaea L.) through the Generation of Subtractive Libraries
Source: Front Plant Sci. 2017 Sep 13;8:1576. doi: 10.3389/fpls.2017.01576 (PMC5601413; doi:10.3389/fpls.2017.01576)

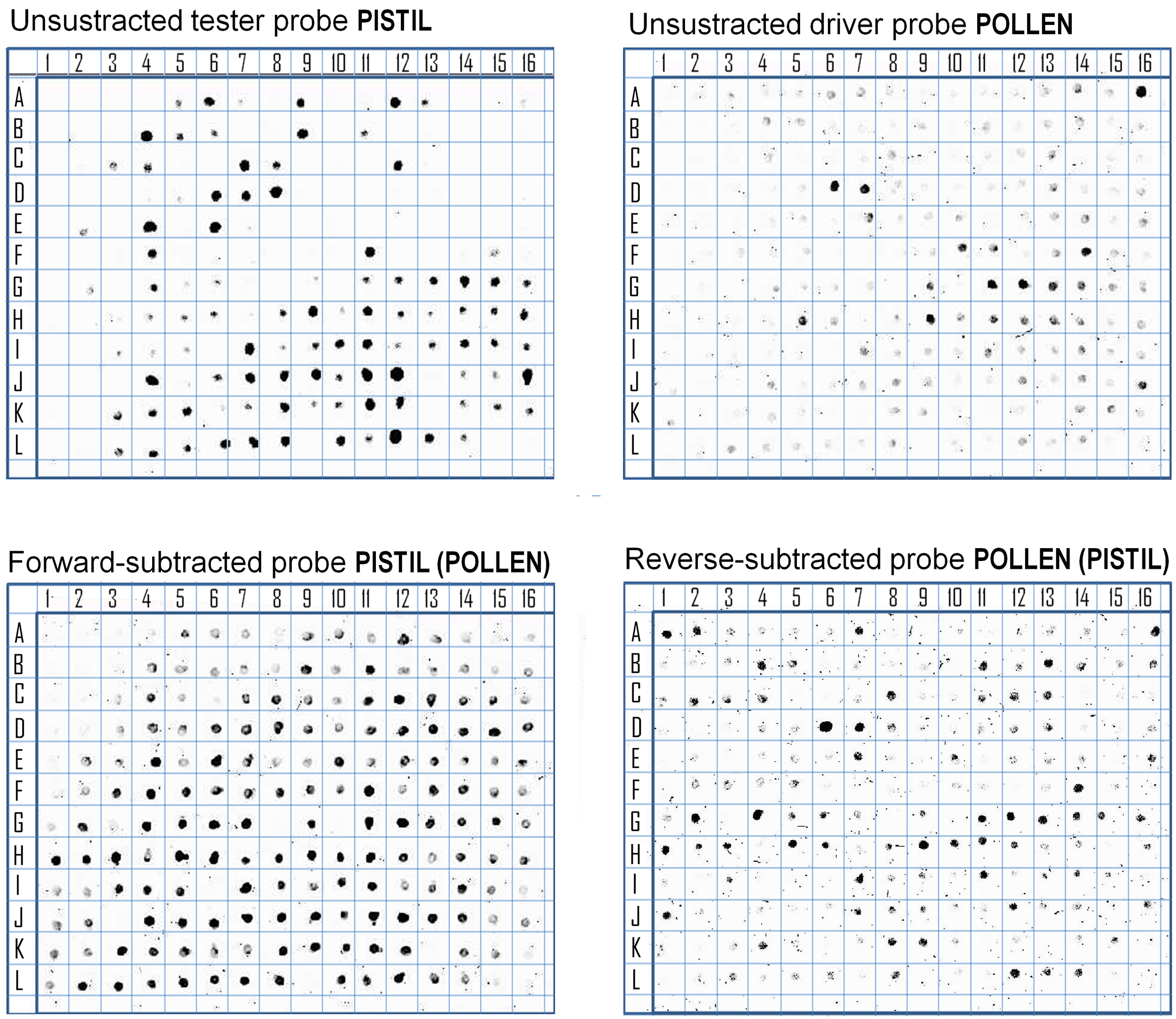

Supplement: Supplementary file 14 [file Image1.TIF]
